# Tracking the Metabolites of Health and Disease Using Artificial Intelligence

**DOI:** 10.3390/diseases14030085

**Published:** 2026-02-25

**Authors:** Ahmed Fadiel, Kenneth D. Eichenbaum, Aya Hassouneh, Kunle Odunsi

**Affiliations:** 1 Computational Oncology Unit, University of Chicago Medicine Comprehensive Cancer Center, 900 E 57th St, KCBD Bldg., Chicago, IL 60637, USA; 2 Department of Anesthesiology, Oakland University William Beaumont School of Medicine, Rochester, MI 48309, USA; keichenbaum@wayne.edu; 3 Department of Anesthesiology, Wayne State University, Detroit, MI 48341, USA; 4 Electrical and Computer Engineering, Western Michigan University, 1903 W. Michigan Ave., Kalamazoo, MI 49008, USA; aia.hassouneh@gmail.com; 5 University of Chicago Medicine Comprehensive Cancer Center, 5841 South Maryland Avenue, MC1140, Chicago, IL 60637, USA; odunsia@uchicago.edu; 6 Department of Obstetrics and Gynecology, University of Chicago, Chicago, IL 60637, USA

**Keywords:** metabolomics, artificial intelligence, multi-omics, cancer, bioinformatics, drug development pipelines

## Abstract

Using AI to analyze metabolite profiles provides critical insights into health, aging, and disease. Metabolomic signatures reveal how lifestyle and therapy impact organ function and cancer progression. This review highlights emerging toolkits for high-throughput data analysis, emphasizing their integration with other omics. Advanced AI approaches facilitate metabolic pathway mapping and accelerate biomarker discovery. By combining AI with multi-omics, researchers can optimize interventions and enhance precision medicine. This article serves as a resource demonstrating AI’s potential in diagnostics and drug discovery.

## 1. Introduction & Background

Metabolomics is the study of how cells process materials into end products. It involves the systematic analysis of molecular intermediates and small products weighing less than 1500 Da^1^. Generating comprehensive datasets allows machine learning to identify molecular patterns in health and disease states. This review provides an overview of the workflow engines used in translational research.

A wealth of information can be collected and accessed to characterize further key information housed inside the metabolome for targeted and untargeted data. Global untargeted data harvesting can be designed to identify as many metabolites as possible within a given matrix [1,2,3,4]. Over time, by tracking metabolite profiles, distinct organ systems can be evaluated, and a composite picture using biomarkers that can provide information about which exposures impact and improve or harm an organ system. With advances in instrumentation and analytical toolkits, it is now possible to develop metabolomic signature patterns that describe physiological and pathological conditions across the spectrum from healthy individuals to patients with advanced disease. While LC–MS is the most commonly used platform in AI-driven metabolomics studies due to its broad analytical versatility and sensitivity across a range of polar and non-polar compounds, other platforms such as GC–MS and CE–MS offer complementary advantages that are underutilized in many AI workflows. LC–MS has become widely adopted because it can separate and detect a wide spectrum of chemical species without requiring chemical derivatization. It also provides high sensitivity in complex biological matrices, making it suitable for many metabolomics applications [5]. In contrast, GC–MS excels at separating and identifying volatile and thermally stable metabolites, often with extensive spectral libraries and reproducible fragmentation patterns, thereby enhancing confidence in the identification of small molecules [6]. Capillary electrophoresis–mass spectrometry (CE-MS) provides high-resolution separation for charged and highly polar compounds that are difficult to retain or separate on conventional LC columns, making it particularly effective for amino acids, nucleotides, and other ionogenic metabolites [7]. Each of these platforms also presents unique analytical challenges that impact downstream AI/ML analyses. For example, LC–MS is susceptible to matrix effects and ion suppression arising from co-eluting endogenous compounds, which can alter ionization efficiency and complicate quantitation [8]. These technical factors can introduce non-biological variability that machine learning models may misinterpret as biological signal if not properly controlled. A critical understanding of these platform-specific limitations is essential for interpreting metabolomic patterns accurately and for designing AI-driven workflows that are both robust and biologically meaningful.

Importantly, these metabolomic signatures can inform drug discovery and development. By identifying disease-specific metabolites and altered pathways, researchers can uncover potential drug targets, evaluate the biochemical effects of candidate therapeutics, and prioritize compounds for further development. Integrating metabolomic data with AI and machine learning enhances the ability to predict drug efficacy, potential toxicity, and mechanisms of action, thereby accelerating the translation of basic metabolic insights into therapeutic interventions.

While high-dimensional metabolomics datasets combined with AI/ML offer powerful pattern recognition and discovery capabilities, translating these approaches into clinical or real-world settings remains challenging. Standardization and reproducibility issues, including differences in instrumentation, sample handling, and analytical protocols, can lead to inconsistent metabolite identification, quantitation, and poor reproducibility across studies, which limit clinical utility and biomarker validation efforts [9]. Comprehensive metabolome analysis often relies on expensive, high-end instruments (e.g., LC–MS) with complex workflows that require significant technical expertise, while routine clinical laboratories typically depend on robust, targeted assays with simpler instrumentation and standardized protocols [10]. Moreover, the lack of broadly accepted best practices for untargeted metabolomics, variability in study design and data processing, and limited validation across diverse cohorts present additional barriers to implementation in precision medicine and clinical diagnostics [11]. Addressing these practical and translational considerations, which include cost, reproducibility, standardization, and validation, can help achieve the full potential of AI-driven metabolomics in precision medicine and diagnostic applications.

Ultimately, tracking metabolomic changes can serve as a dynamic monitor for organ wellness, systemic health, and treatment response. Over time, applying artificial intelligence (AI) to these molecular patterns not only improves disease understanding but also supports the discovery and optimization of new therapies.

### Recent Advances in AI-Driven Metabolic Pathway Modeling

Recent years have witnessed a rapid expansion of deep learning methodologies for metabolomics data interpretation, particularly in the prediction of metabolic pathways and flux distributions [12]. Since 2023, transformer-based architectures, graph neural networks (GNNs), and variational autoencoders (VAEs) have been increasingly applied to model complex biochemical reaction networks and to infer pathway activity directly from high-dimensional metabolomic profiles. These approaches enable the identification of latent metabolic states and improve the resolution of pathway-level biomarker discovery beyond traditional multivariate statistics. Graph neural networks have shown particular promise for pathway inference by representing metabolites as nodes and enzymatic reactions as edges, thereby allowing biologically constrained learning of metabolic network topology and activity [13,14]. Recent studies have demonstrated that GNN-based metabolomics models can accurately predict pathway dysregulation in cancer metabolism, mitochondrial disorders, and inflammatory diseases, outperforming conventional enrichment-based approaches. Transformer models have also been adapted for metabolomics to capture long-range dependencies among metabolites and to predict metabolic flux alterations under pharmacological or nutritional perturbations.

Importantly, these deep learning strategies are increasingly being integrated into drug discovery pipelines, enabling in silico prioritization of metabolic enzymes as therapeutic targets, prediction of off-target metabolic liabilities, and patient stratification based on pathway activity signatures. The integration of AI-based pathway modeling with multi-omics data is thus accelerating translational research by bridging metabolomic pattern recognition with mechanistic interpretation and therapeutic development.

In this review, we apply a hierarchical framework for computational analysis: Artificial Intelligence (AI) serves as the broad field of systems mimicking human intelligence; Machine Learning (ML) refers to the specific algorithms that learn from embedded data to identify patterns without explicit programming; and Deep Learning (DL) is a specialized subset of ML that utilizes multi-layered neural networks to manage the most complex, high-dimensional datasets.

## 2. Metabolomics Landscape and Foundational Research

There are many emerging opportunities for metabolomics to investigate pathological disorders in the context of small molecules. This growing field, like proteomics and genomics, can stitch together different molecular cascades that help characterize how metabolites form and are utilized in complex biological systems and disease processes. Wishart’s review [15] discussed several recent technological developments in metabolomics, emphasizing how to use metabolomics to find the root causes of complicated disorders. Pang and coworkers [16] highlighted developments in metabolomics technology and methodologies and how they apply to the study of clinical pharmacology. They addressed many of the field’s present difficulties and prospective future paths. Yang and coworkers highlighted new applications of metabolomics biotechnology in revealing metabolic anomalies and related underlying disease mechanisms [17]. The application of metabolomics to cancer and chemotherapy assessment was reported by Gao et al. [18]. They highlighted the importance of metabolomics in mass spectrometry applications and in clinical and laboratory gastrointestinal cancer investigations. Tumor growth can stem from both interwoven epigenetic and metabolomic changes. Metabolites have experimentally been shown to directly modulate chromatin factors and epigenetic modifications alter transcriptional regulation of metabolic enzymes leading to metabolic reprogramming [19,20].

Fu et al. [21] performed a metabolomics analysis to review the study findings on the interventional effects of functional meals on metabolic disorders. These analyses demonstrate the wide range of applications for metabolomics that include animal model preclinical studies, drug screening, drug efficacy and toxicity studies, and comprehensive clinical evaluation. Collectively, these studies illustrate how metabolomics can inform drug screening, evaluate drug efficacy and toxicity, and guide the development of novel therapeutics.

Our understanding of the mechanisms of molecules linked to many metabolic illnesses is at an early stage. Targeting precise downstream effects of functional biologics, genetic targeting, and dietary interventions on metabolic illnesses can be enhanced using metabolomics with other “omics” technologies. The swift development of metabolomic techniques has made it possible to quickly detect and identify tiny molecules in biological and environmental materials. These tools are increasingly capable of uncovering novel metabolites and pathways that can serve as potential drug targets, inform mechanism-of-action studies, and accelerate therapeutic discovery pipelines. Kaddurah-Daouk and co-workers [22] emphasized the use of metabolomics in the analysis of nervous system disorders. They share ideas on metabolomics, metabolic profiling technologies, preliminary research on neuropsychiatric disorders, and medications used to treat these disorders. Increasing usage of metabolomics in medicine facilitates the identification of biomarkers for diagnosing illness and treatment, and therapeutic intervention design, as discussed by the review of Aderemi and coworkers [23]. Recently, Abdelhamid and coworkers discussed a machine learning algorithm analysis of 8500 biomarkers using metabolomics, proteomics, and lipidomic to predict survival outcomes in trauma patients [24], demonstrating how AI-driven multi-omics analysis can prioritize targets and guide drug development strategies.

## 3. Inside the Metabolomics Workflow Engine: Software Tools

**Figure 1:**
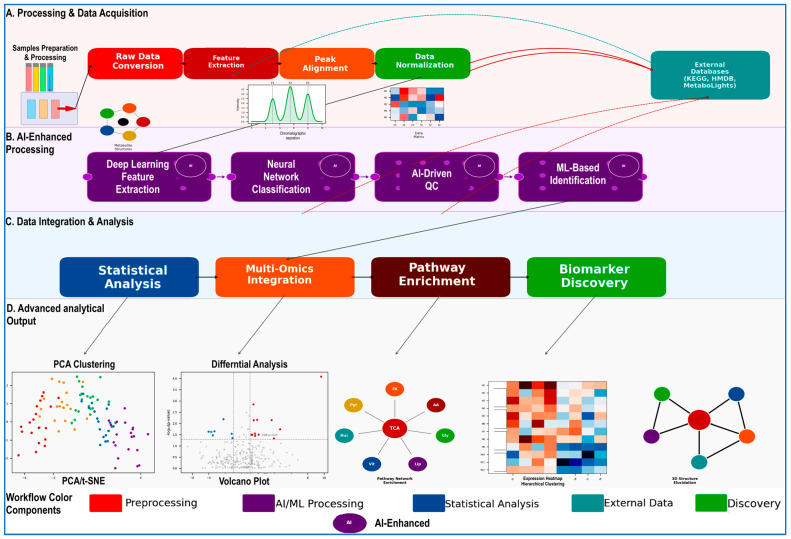
Integrated metabolomics analysis pipeline combining conventional preprocessing with artificial intelligence (AI) and machine learning (ML)–assisted analysis. (**A**) Preprocessing and data acquisition: Biological samples are extracted and analyzed by liquid chromatography–tandem mass spectrometry (LC–MS/MS). Chromatographic separation generates retention time–resolved ion signals that are converted into structured data matrices (*m*/*z*–retention time features). Subsequent steps include raw data conversion, feature extraction (peak detection), retention time alignment, and normalization. External databases such as HMDB, KEGG, and MetaboLights support metabolite annotation and biological interpretation. (**B**) AI/ML-assisted processing layer: This layer comprises four interconnected computational modules (purple boxes): representation learning (e.g., deep learning–based feature extraction), classification (e.g., supervised models), data quality control that includes outlier detection and batch correction, and ML-assisted metabolite annotation. These modules enhance robustness and scalability while supporting reproducible downstream analysis. (**C**) Data integration and analysis: Statistical analysis includes univariate and multivariate approaches with appropriate multiple-testing correction (e.g., false discovery rate [FDR]). Data integration incorporates correlation networks and data fusion across complementary datasets such as transcriptomics or proteomics where available. Pathway enrichment analysis uses curated resources that may include KEGG, Reactome, and WikiPathways to contextualize metabolic alterations. Biomarker discovery applies feature selection and receiver operating characteristic (ROC) analysis with appropriate validation strategies. Red arrows indicate external database queries and iterative annotation refinement. (**D**) Analytical outputs and disease-relevant interpretation: Outputs include dimensionality reduction such as principal component analysis [PCA] and t-distributed stochastic neighbor embedding [t-SNE] as well as volcano plots for differential abundance analysis (FDR-adjusted *p* < 0.05; |log_2_ fold change| > 1). Pathway network visualization highlights the central role of the tricarboxylic acid (TCA) cycle and its connections to glycolysis, amino acid metabolism, lipid metabolism, nucleotide metabolism, and cofactor/vitamin pathways. Additional outputs include hierarchical clustering heatmaps and molecular structure annotation or elucidation that are supported by spectral evidence. AI/ML-assisted components improve analytical scalability and support reproducible identification of disease-associated metabolic signatures while maintaining rigorous statistical control. Color coding: red/coral (preprocessing), purple (AI/ML processing), blue (statistical analysis), orange (data integration), brown (pathway analysis), green (biomarker discovery), teal (external databases).

Software tools designed specifically for metabolomics data are rapidly being developed, with hundreds of tools already available in the literature. Many of these tools are open-source and freely available. Metabolomic tools offer the advantages of being fast, cheap, and sensitive. However, the data generated by these tools mean little unless they are adequately analyzed for the construction of biochemical pathways and for understanding how they interact in both diseased and non-diseased states. A full Metabolomic path encompasses the workflow process spanning preprocessing, data collection, and postprocessing and is illustrated in Figure 1. Initially, the biological sample is processed, in which molecules of interest are extracted and any interfering substances are removed. Adaptive sampling to determine improved resolution for scanning and optimized LC-MS settings can be introduced with AI techniques. Raw data must be converted to a specific format, often open source, to be accepted by the platform tools. Then, the data will be processed using those tools to find the Peak, or unique identifying feature, that stands out from background noise and is then aligned and normalized. Alignment allows for matching the same molecule across datasets. The samples can be corrected for unwanted variation, adjusting for concentration and setting up internal standards with quality control tools. Software can compare peaks against external datasets. In-silico prediction can be applied to identified peaks. Once the Dataset is organized, principal component analysis can be used to see which data clusters are found together with the help of graphical neural networks. Additional analysis can identify changes and pathological aberrancies with increases and decreases in certain molecules.

Commercial metabolomics platforms have transformed mass spectrometry-based metabolomics workflows by integrating data-processing algorithms into vendor-specific, user-friendly software environments [4,15]. Platforms such as MassProfiler Professional, Compound Discoverer, PeakView, MarkerLynx, MetaboScape, and Profiler AM+ (Table 1) deliver end-to-end solutions for handling raw data, peak detection, feature alignment, normalization, statistical modeling, and metabolite annotation [25,26]. These systems have modernized metabolomics capabilities for laboratories lacking dedicated bioinformatics infrastructure [27].

The principal strength of commercial platforms lies in their seamless integration with specific instrument ecosystems, enabling management of large-scale datasets with stable file-format support and access to continuously updated spectral libraries and vendor technical assistance [28,29]. Guided workflows and intuitive graphical interfaces facilitate standardization and implementation of quality-control procedures, which are requisite for high-throughput studies and regulated research environments [30]. These features are valuable in clinical metabolomics and pharmaceutical applications where reproducibility and regulatory compliance are mandated [31,32].

There are several limitations that constrain universal applicability for commercial platforms. Licensing and maintenance costs can be onerous and may impede access in academic and developing-world settings [33,34]. The proprietary nature of underlying algorithms and data structures presents challenges for transparency and independent validation, often complicating efforts to reproduce published analytical workflows [35,36]. Vendor-specific data formats create “data lock-in” scenarios that impede integration with outside software ecosystems and obstruct long-term cross-platform analysis or multi-omics integration [37,38]. For custom experimental designs, limited flexibility may prevent implementation of bespoke statistical models or cutting-edge machine-learning approaches [39,40].

Some metabolomics laboratories have adopted hybrid analytical strategies that involve using commercial platforms for data acquisition, initial preprocessing, and quality control, while exporting curated feature tables and spectral data to open-source environments [41,42]. This allows for transparent, extensible downstream analysis. Balancing the reliability of commercial tools with the algorithmic transparency and flexibility of community-developed software enables reproducibility, interoperability, and sustainable data stewardship in metabolomics research [43,44].

The approach to generating data with metabolomics requires a preprocessing assessment of which platform and database tools (Table 2 and Table 3) should be used: NMR-based, MS-based, or FT-IR Spectroscopy-based. A recently designed software website: MSCAT (Metabolomics Software CATalog) database of metabolomics software tools, provides an overview of available tools and assists researchers in choosing a data analysis workflow for metabolomics studies according to their specific needs [45]. MSCAT’s database can be used to identify the output of the preprocessing tools. There are several approaches to data harvesting in metabolomics, using either mass spectrometry (MS), nuclear magnetic resonance (NMR) spectroscopy, or Fourier-transform infrared (FTIR) spectroscopy techniques. Examples of such approaches include metabolomic fingerprinting, metabolic profiling, metabolic foot printing, target analysis, and flux analysis, each playing significant roles in understanding toxicological mechanisms and disease processes in living organisms and identifying potential therapeutic targets for drug discovery. Platform technologies such as Nuclear Magnetic Resonance have been used specifically in applications to detect novel chemicals and clarify structures. Mass Spectrometry allows for highly sensitive separation techniques and is becoming more widely used.

**Table 1:** Commercial Metabolomics Software Platforms: Comparative Overview.

Platform	Vendor	Primary Functions	Key Strengths	Notable Limitations	Target Instruments
MassProfiler Professional	Agilent	Chemometric analysis, feature extraction, multivariate statistics, pathway mapping	Robust Agilent integration, comprehensive statistical visualization, database-linked annotation	Limited cross-vendor compatibility, closed algorithmic architecture	Agilent GC/MS, LC/MS, CE/MS
Compound Discoverer	Thermo Fisher	Untargeted/semi-targeted workflows, preprocessing, library identification (mzCloud, mzVault)	Node-based workflow design, extensive library ecosystem, multi-modal MS support	Premium licensing costs, restricted extensibility	Thermo Fisher LC–MS, high-resolution MS
PeakView	SCIEX	Qualitative LC–MS/MS analysis, spectral visualization, structural elucidation	Intuitive spectrum-centric interface, batch processing capabilities	Primarily qualitative focus, SCIEX-specific formats	SCIEX TripleTOF, LC–MS/MS systems
MarkerLynx XS	Waters	Automated feature detection, data matrix generation, multivariate analysis (PCA)	Optimized Waters UPLC–MS integration, robust peak alignment	Proprietary environment, limited external interoperability	Waters UPLC–MS (MassLynx)
MetaboScape	Bruker	Discovery metabolomics/lipidomics, T-ReX feature extraction, CCS-aware workflows	Advanced multidimensional visualization, ion-mobility integration	Optimized for Bruker formats, costly library licensing	Bruker high-resolution, ion-mobility MS
Profiler AM+	Shimadzu	LC/MS, GC/MS analysis, metabolic pathway mapping, quantitative visualization	Seamless method package integration, metabolic map visualization	Shimadzu-centric ecosystem, limited algorithmic transparency	Shimadzu LC–MS, GC–MS platforms

Table adapted and synthesized from vendor documentation and published platform comparisons [25,29,46].

**Table 2:** Overview of Software Tools.

Tool Name	Type	Platform	Dependency/Implementation	Software Availability	Validation Reference (Example)	Performance Metrics (Appropriate Wording)	Typical Use Cases
XCMS Online	GUI	Web	Cloud-hosted web application; backend built on R/XCMS with custom web services and job scheduling	https://xcmsonline.scripps.edu (accessed on 15 September 2023)	Smith et al., 2006; Tautenhahn et al., 2012 [47,48]	Performance varies by dataset and parameters; validated for reproducible feature detection across studies	Peak detection, retention time alignment, comparative metabolomics
MZmine	GUI	Cross-platform	Java (platform-independent); supports mzML, mzXML, NetCDF; modular processing pipeline	http://mzmine.github.io (accessed on 15 September 2023)	Pluskal et al., 2010 [49]	No single sensitivity metric; benchmarked for comprehensive feature detection and visualization	End-to-end LC–MS data processing and visualization
MetaboAnalyst	GUI	Web	R backend with Shiny web framework; relies on multiple CRAN/Bioconductor packages	https://www.metaboanalyst.ca (accessed on 15 September 2023)	Chong et al., 2019 [50]	Statistical validity depends on selected normalization and models; widely benchmarked	Statistical analysis, pathway analysis, metabolite profiling
MS-DIAL	GUI	Windows	C# (.NET Framework); includes in-house spectral libraries; not VBA-dependent	http://prime.psc.riken.jp/Metabolomics/MS-DIAL (accessed on 15 September 2023)	Tsugawa et al., 2015 [42]	High coverage and annotation rate for untargeted LC–MS/MS in benchmarking studies	Non-targeted LC–MS/MS, spectral deconvolution, compound annotation
XCMS	R package	Cross-platform	R with C/C++; Bioconductor package; integrates MSnbase, BiocParallel	https://bioconductor.org/packages/xcms (accessed on 15 September 2023)	Smith et al., 2006; Tautenhahn et al., 2008 [47,51]	Robust and reproducible feature detection when parameters are optimized	Peak picking, retention time correction, alignment
RAMClustR	R package	Cross-platform	Pure R implementation; operates downstream of XCMS/MZmine outputs	https://cran.r-project.org/package=RAMClustR (accessed on 15 September 2023)	Broeckling et al., 2014 [52]	Demonstrated improvement in feature clustering coherence (no universal accuracy metric)	Spectral feature clustering into compounds
CAMERA	R package	Cross-platform	R (Bioconductor); designed to work with XCMS objects	https://bioconductor.org/packages/CAMERA (accessed on 15 September 2023)	Kuhl et al., 2012 [53]	Evaluated for isotope/adduct annotation performance	Peak annotation, isotope and adduct grouping
MetAlign	GUI	Windows	C++; historically 32-bit executable (runs on 64-bit Windows); optimized for large datasets	https://www.plantmetabolomics.org/metalign (accessed on 15 September 2023)	Lommen, 2009 [54]	Demonstrated high alignment sensitivity in GC–MS and LC–MS datasets	Signal alignment, normalization
MetaboLyzer	R package	Cross-platform	R; integrates statistical testing and metabolite identification workflows	https://cran.r-project.org/package=MetaboLyzer (accessed on 15 September 2023)	Mak et al., 2013 [55]	Emphasis on statistical robustness rather than raw detection sensitivity	Differential metabolomics, statistical analysis

**Table 3:** Comprehensive List of Database Tools.

Software Tool	Platform Dependency	Implementation	Software Availability	Ref.
COCONUT	Vendor-neutral (open formats); Cloud-based	Web	https://coconut.naturalproducts.net (accessed on 15 September 2023)	[56]
MUTLIN MS2 Molecular standards database	LC-MS/MS	Web	http://metlin.scripps.edu/ (accessed on 15 September 2023)	[57]
CSMDB	NMR	MATLAB R2027b	https://github.com/cibionnmrlab/CSMDB-with-ConQuer-ABC (accessed on 15 September 2023)	[58]
EMBL-MCF	LC-MS	NA	https://curatr.mcf.embl.de/ (accessed on 15 September 2023)	[59]

Table 4 illustrates the advantages and disadvantages of standard metabolomics tools. Available software tools are listed in sequential order in characterizing a metabolomics study. First metabolomic tools are used on samples to obtain data and results are generated by the instruments. Then, output data is preprocessed (Table 5) and investigated using quality control tools (Table 6). Subsequently, annotation tools (Table 7), are used to define the success and failure of untargeted metabolomics. Finally, statistical and visualization tools (Table 8) are used to plot metabolite data sets, convert, and clean up the data. Additionally, these instruments conduct data normalization for sample labeling and list metabolite names within the experimental conditions. These workflows can also be integrated with AI to accelerate biomarker discovery and identify druggable metabolic pathways, bridging basic metabolomics insights to therapeutic development (Table 9 and Table 10).

**Table 4:** Advantages and Disadvantages of Standard Metabolomics Tools.

Platform	Strengths	Weaknesses	Sensitivity Rating (1–5) *	Library Availability Rating (1–5) *
XCMS Online	User-friendly web interface; robust peak detection	Limited to web access; server dependency	4	5
MZmine	Comprehensive feature set; good visualization tools	Requires Java installation	4	4
MetaboAnalyst	Extensive statistical and visualization modules	Web-based; requires stable internet connection	5	5
MS-DIAL	Strong for non-targeted LC–MS/MS workflows	Windows-only desktop application	4	4
XCMS	Well-established peak picking and alignment	Steeper learning curve; R scripting needed	4	4
RAMclustR	Effective clustering of features into spectra	Requires R proficiency	4	3
CAMERA	Powerful peak annotation (isotopes, adducts)	Limited to R environment	4	4
MetAlign	Accurate alignment and baseline correction	Windows-only GUI; legacy interface	4	4
MetaboLyzer	Supports metabolite identification and statistics	Requires R knowledge; more limited ecosystem	3	3

* Ratings are qualitative and provided for comparative illustration across commonly used platforms, not as absolute performance measures.

**Table 5:** Comprehensive List of Reviewed Preprocessing Tools.

Software Tool	Platform Dependency	Implementation	Software Availability	Ref.
CROP	LC–MS/MS	R 3.5.3	https://github.com/rendju/CROP (accessed on 15 September 2023)	[60]
ncGTW	LC–MS/MS	R 3.5.3, C++	https://github.com/ChiungTingWu/ncGTW (accessed on 15 September 2023)	[61]
TidyMS	LC–MS/MS	Python 3.8	https://github.com/griquelme/tidyms (accessed on 15 September 2023)	[62]
AutoTuner	LC–MS/MS	R 4.0.1	https://github.com/crmclean/Autotuner (accessed on 15 September 2023)	[63]
hRUV	LC–MS/MS	R 4.1.0	https://shiny.maths.usyd.edu.au/ hRUV/ (accessed on 15 September 2023)	[64]
MetumpX	Any	R 3.5.3	https://github.com/hasaniqbal777/MetumpX-bin (accessed on 15 September 2023)	[65]

**Table 6:** Comprehensive List of Reviewed QC Tools.

Software Tool	Platform Dependency	Implementation	Software Availability	Ref.
MetaQuac	Targeted LC–MS	R 3.4.4	https://github.com/bihealth/metaquac (accessed on 15 September 2023)	[65]
dbnorm	Vendor-neutral (open formats); Desktop (local)	R 4.0.1	https://github.com/NBDZ/dbnorm (accessed on 15 September 2023)	[66]
MetaClean	LC–MS/MS	R 4.1.0	https://cran.r-project.org/src/contrib/Archive/MetaClean/ (accessed on 15 September 2023)	[67]
NeatMS	LC–MS/MS	Python 3.8	https://github.com/bihealth/ (accessed on 15 September 2023)	[68]

**Table 7:** Comprehensive List of Reviewed Annotation Tools.

Software Tool	Platform Dependency	Implementation	Software Availability	Ref.
MESSAR	LC–MS/MS	Web	https://messar.biodatamining.be/ (accessed on 15 September 2023)	[69,70]
SMART 2.0	2D NMR	Web	https://smart.ucsd.edu/classic (accessed on 15 September 2023)	[71]
MetFID	MS/MS data	Python-based artificial neural network (ANN) model implemented using Keras/TensorFlow	Method described in literature; no dedicated public web server reported	[72]
CPVA	Vendor-neutral (open formats); Cloud-based	Web	https://academic.oup.com/bioinformatics/article/36/12/3913/5809525 (accessed on 15 September 2023)	[73]
NRPro	LC–MS/MS	Java, Web	https://bioinfo.cristal.univ-lille.fr/nrpro/ (accessed on 15 September 2023)	[74]
MetENP/MetENPWeb	LC–MS/MS	R, Web	https://www.metabolomicsworkbench.org/data/analyze.php (accessed on 15 September 2023)	[75]
CANOPUS	LC–MS/MS	Standalone	https://bio.informatik.uni-jena.de/software/canopus/ (accessed on 15 September 2023)	[71]
MolDiscovery	LC–MS/MS	Python 3.8	https://github.com/mohimanilab/molDiscovery (accessed on 15 September 2023)	[76]
MetIDfyR	LC–MS/MS	R 4.0.0	https://github.com/agnesblch/MetIDfyR (accessed on 15 September 2023) MetIDfyR	[77]
Qemistree	LC–MS/MS	Python 3.8	https://github.com/biocore/q2-qemistree (accessed on 15 September 2023)	[78]
IIMN	LC–MS/MS	GNPS, Web	https://ccms-ucsd.github.io/GNPSDocumentation/fbmn-iin/ (accessed on 15 September 2023)	[79]
FOBI	Vendor-neutral (open formats); Hybrid (cloud + local)	R, Web	https://github.com/pcastellanoescuder/FOBI_Visualization_Tool (accessed on 15 September 2023)	[80]
Biodendro	LC–MS/MS	Python 3.8	https://github.com/ccdmb/BioDendro (accessed on 15 September 2023)	[81]
AllCCS atlas	IM-MS	Web	https://github.com/ZhuMetLab/ AllCCS; http://allccs.zhulab.cn/ (accessed on 15 September 2023)	[82]
Binner	LC–MS/MS	Java	https://binner.med.umich.edu/ (accessed on 15 September 2023)	[83]
MS-CleanR	LC–MS/MS	R <=4.2	https://github.com/eMetaboHUB/MS-CleanR (accessed on 15 September 2023)	[84]
Retip	LC–MS/MS	R 4.0.0	https://www.retip.app/ (accessed on 15 September 2023)	[85]
QSRR Automator	LC–MS/MS	Python 3.8	https://github.com/UofUMetabolomicsCore/QSRR_Automator/releases/tag/v1_exe (accessed on 15 September 2023)	[86]
MFAssignR	LC–MS/MS	R, HTML	https://github.com/skschum/MFAssignR (accessed on 15 September 2023)	[87]
McSearch	LC–MS/MS	R 4.0.0	https://github.com/HuanLab/McSearch (accessed on 15 September 2023)	[88]
REDU	LC–MS/MS	GNPS, Web	https://ccms-ucsd.github.io/GNPSDocumentation/ReDU/ (accessed on 15 September 2023)	[89]
MASST	LC–MS/MS	GNPS, Web	https://masst.gnps2.org/ (accessed on 15 September 2023)	[90,91]
NPClassifier	Any	Web	https://npclassifier.ucsd.edu/ (accessed on 15 September 2023)	[92]
patRoon	HR MS/MS	R 4.3.3	https://github.com/rickhelmus/patRoon (accessed on 15 September 2023)	[93]
LipidLynxX	LC–MS/MS	Python 3.8, Standalone	http://lipidmaps.org/lipidlynxx/ (accessed on 15 September 2023)	[94]

**Table 8:** Comprehensive List of Reviewed Statistical and Visualization Tools.

Software Tool	Analysis Type/Optimization	Platform Dependency	Implementation	Software Availability	Ref.
Epimetal	Visualization/Exploration	Vendor-neutral (open formats); Cloud-based	JavaScript, Web	https://github.com/amergin/epimetal; https://github.com/amergin/epimetal (accessed on 15 September 2023)	[95]
Metabolite AutoPlotter	Quantitative/Targeted	Quantitative metabolomics data, any	R, Web	https://mpietzke.shinyapps.io/AutoPlotter/ (accessed on 15 September 2023)	[96]
Metabolite-Investigator	Quantitative/Targeted	LC-MS	R, Web	https://github.com/cfbeuchel/Metabolite-Investigator (accessed on 15 September 2023)	[97]
VIIME	Visualization/Exploration	Vendor-neutral (open formats); Cloud-based	Web	https://viime.org/#/ (accessed on 15 September 2023)	[98]
struct	Quantitative/Targeted	Vendor-neutral (open formats); Desktop (local)	R based	https://bioconductor.org/packages/release/bioc/html/struct.html (accessed on 15 September 2023)	[99]
lipidr	Untargeted LC-MS	LC-MS/ MS	R based	https://www.lipidr.org/ (accessed on 15 September 2023)	[100]
NOREVA (Statistics Only)	Quantitative/Statistical Analysis	Vendor-neutral (open formats); Hybrid (cloud + local)	Web, R, Standalone	http://idrblab.cn/noreva/ (accessed on 15 September 2023)	[101]
%polynova_2way (Statistics Only)	Quantitative/Statistical Analysis	Processed Data	SAS	https://doi.org/10.1371/journal.pone.0244013 (accessed on 15 September 2023)	[102]
rawR (Visualization)	Visualization/Exploration	LC-MS	R 4.0.0, C++	https://github.com/fgcz/rawR (accessed on 15 September 2023)	[103]
MEtaboverse (Visualization)	Visualization/Exploration	Vendor-neutral (open formats); Desktop (local)	Java, HTML, Stanalone	https://github.com/Metaboverse (accessed on 15 September 2023)	[104]
JS-MS 2.0 (Visualization)	Visualization/Exploration	LC-MS/MS	Java, JavaScript, HTML	https://github.com/optimusmoose/jsms (accessed on 15 September 2023)	[105]
Metpropagate (Visualization)	Untargeted LC-MS	Untargeted LC-MS	R package, Python > 3.4	https://github.com/emmag (accessed on 15 September 2023)	[106]

**Table 9:** Comprehensive List of Isotopic Tools.

Software Tool	Platform Dependency	Implementation	Software Availability	Ref.
isoSCAN	GC-MS	R package	https://github.com/jcapelladesto/ isoSCAN (accessed on 15 September 2023)	[107]
MIAMI	GC-MS	Compiled C++ source code	https://md.tu-bs.de/ (accessed on 15 September 2023)	[108]

**Table 10:** Comprehensive List of Lipidomics, MSI and Multiomics Tools.

Software Tool	Platform Dependency	Implementation	Software Availability	Ref.
LiPydomics	Lon Mobility, Lipidomics	Python, HTML	https://github.com/dylanhross/lipydomics (accessed on 15 September 2023)	[109]
LipidCreator	LC-MS	C#, HTML, Skyline plugin	https://github.com/lifs-tools/lipidcreator (accessed on 15 September 2023)	[110]
Lipid Annotator	LC-MS/MS	Raman imaging/MSI (LC-MS/MS imaging)	R and C++ (open-source R package)	[111]
Raman2imzML	Raman	C++, R package	https://github.com/LlucSF/Raman2imzML (accessed on 15 September 2023)	[112]
SUMMER	LC-MS/Lipidomics	R, Web	http://igc1.salk.edu:3838/summer/ and https://bitbucket.org/salkigc/summer/src/master/ (accessed on 15 September 2023)	[112]

The software tools detailed in Table 1, Table 2, Table 3, Table 4, Table 5, Table 6, Table 7, Table 8 and Table 9 do not function in isolation but comprise a synchronized ‘metabolomics workflow engine.’ For example, the MSCAT database serves as more than a catalog; it is an intelligent assistant that helps researchers choose a specific data analysis workflow tailored to the unique sensitivities of MS or NMR platforms. By integrating these tools, researchers can ‘stitch together’ different molecular cascades, transforming raw data into a composite picture of organ wellness and systemic health.

## 4. Analysis of Metabolomic Data Using Machine Learning and Artificial Intelligence

**Figure 2:**
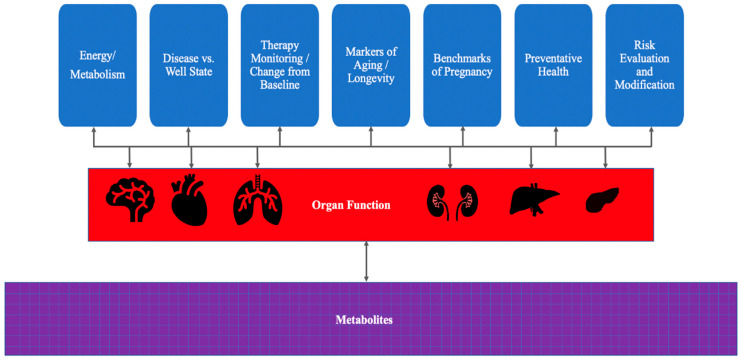
Developing Metabolomics Applications in Health and Disease.

Machine learning algorithms are required to map disease states from complex datasets. These systems learn from embedded data to identify unique patterns without explicit programming. Deep learning models provide opportunities to individualize diagnosis and predict drug responses. This facilitates the advancement of precision medicine and therapeutic discovery. Furthermore, integrating pathway predictions with AI helps researchers identify drug targets and off-target effects. Sampling data from healthy and diseased individuals can distinguish between the two groups based on the patterns of metabolites present (Figure 2). It is important to distinguish the type of insight AI provides: most applications identify statistical associations between metabolites and phenotypes, some models predict pathway membership using network-based approaches, and others can generate mechanistic hypotheses, which require experimental validation. For example, researchers can use metabolomic data to map complications during pregnancy [113] and monitor solid tumor progression throughout a treatment course [114].

It is important to distinguish the type of insight AI provides in metabolomics. Most AI applications in this field primarily detect statistical associations; as differentiating healthy from diseased individuals based on metabolite profiles. Some models, particularly graph neural networks and pathway-based deep learning approaches can predict pathway membership and identifying which metabolic routes are likely active or dysregulated. While these predictions can suggest possible intervention points, true mechanistic insight; the causal understanding of how specific metabolites influence biological processes; requires experimental validation. Therefore, AI serves as a powerful tool for pattern recognition and hypothesis generation, but translating these findings into mechanistic understanding relies on complementary biochemical and molecular studies.

Another application of machine learning in metabolomics is the prediction of metabolic pathways and networks that regulate routine homeostasis. Machine learning contributes to pathway and network analysis through pathway enrichment, network inference and hybrid knowledge-driven approaches where prior biochemical knowledge is integrated with high-dimensional metabolomic data to improve pathway prediction and suggest potential mechanistic relationships. By integrating metabolomic pathway predictions with AI, researchers can identify potential drug targets, therapeutic intervention points, and off-target effects, supporting drug development and mechanism-of-action studies. Metabolism involves reactions that convert raw materials into energy and other useful valuable molecules. Understanding these pathways and networks can provide insight into the functioning of cells and tissues. Machine learning algorithms can be used to analyze metabolomics data and infer the underlying pathways and networks, which can help to identify potential targets for therapeutic intervention or to understand the mechanisms of diseases. Assays that measure metabolites for endpoint targeting, such as cardiovascular event risk, trauma outcomes [24], glucose tolerance [115], body composition correlation with health, kidney and liver health [116], and the impact of lifestyle on health, are presently being developed [117,118]. A recent comprehensive review details software and informatics tools specifically dedicated to lipid metabolism and the intricacies of lipidomics [119].

### 4.1. From Sample Data to Intelligent Mapping of Pathways

AI capabilities are categorized by their complexity and application. Machine learning algorithms are utilized to map health states from large datasets and improve analysis methods through automated processing. In contrast, Deep learning networks, such as Convolutional Neural Networks (CNNs) and Recurrent Neural Networks (RNNs), rely on decision trees and neural architectures to develop complex automated detection capabilities, extending from raw data preprocessing to feature selection and taxonomy. Machine learning algorithms are designed for computers to improve data analysis methods without explicit programming. Over time they develop improved accuracy and precision in learning complex patterns, and their automated processing permits improved task-oriented speed and efficiency. They also facilitate the interpretation of results. Deep learning networks rely on neural networks and decision trees to develop complex automated analysis and detection capabilities. They can be implemented into each data harvesting step beginning with data preprocessing and extending to feature selection and taxonomy. Rather than providing a direct map, AI-driven analysis infers pathological states by identifying complex signatures within high-dimensional data. For example, machine learning algorithms have been utilized to analyze 8500 multi-omic biomarkers to predict 30-day survival and recovery in trauma patients. Similarly, AI has been applied to plasma profiles to predict the response to neoadjuvant chemotherapy in triple-negative breast cancer and to detect intrauterine growth restriction by analyzing multi-platform metabolomics data. In renal health, these tools (Table 11) facilitate the prediction of chronic kidney disease progression, moving from general observation to specific prognostic indicators.

### 4.2. Examples of Artificial Intelligence in Metabolomics Data Analysis

#### 4.2.1. Classical Machine Learning Methodologies

Classical approaches involve both supervised methods (using labeled data to predict group membership) and unsupervised methods (identifying patterns without predefined labels).

- Random Forest (Supervised): This algorithm is most appropriate for identifying biomarkers in disease diagnosis due to its high accuracy and robustness against overfitting.
- Support Vector Machine (SVM) (Supervised): SVM is highly effective at classifying samples based on metabolic profiles, particularly when handling high-dimensional data.
- k-Nearest Neighbors (k-NN) (Supervised): This is a simple methodology primarily used for the classification of samples according to their metabolic profiles.
- Linear Discriminant Analysis (LDA) (Supervised): LDA is used for both biomarker identification and sample classification, assuming the data follows a Gaussian distribution.
- Principal Component Analysis (PCA) (Unsupervised): PCA is widely utilized for dimensionality reduction, data visualization, identifying metabolic pathways, and clustering samples.

#### 4.2.2. Deep Learning Methodologies

Deep learning relies on neural networks and decision trees to perform complex automated analysis across various stages of the metabolomics workflow, from preprocessing to feature selection1. These models are specifically used to individualize diagnosis, treatment, and drug response prediction.

- Artificial Neural Networks (ANN)/General Neural Networks: These are most appropriate for identifying metabolic pathways and predicting metabolic fluxes because they can manage very large datasets with high accuracy.
- Convolutional Neural Networks (CNN): Beyond image recognition, CNNs are used for identifying metabolic images, predicting metabolic fluxes, and performing regression and pattern categorization on high-dimensional spectra like MS or NMR.
- Recurrent Neural Networks (RNN): RNNs are specialized for sequential and time-series data, making them ideal for identifying metabolic pathways and predicting fluxes over time4. They are also used to analyze the sequential features of high-dimensional spectra.
- Deep Belief Networks (DBN): These networks excel at unsupervised learning and feature extraction, making them suitable for identifying unknown metabolic compounds and clustering samples.

Organizing biomarker samples into clusters reduces the data dimensionality and permits easier classification. Complex, high-dimensional metabolomics data, such as mass spectrometry or nuclear magnetic resonance spectra, are most effectively analyzed using convolutional neural networks (CNNs) for spatial and spectral feature extraction, whereas recurrent neural networks (RNNs) are specifically applied to sequential metabolomics datasets, including longitudinal, time-course, dynamic fluxomic, and treatment-response studies. Recurrent neural networks are primarily designed to model ordered sequences and temporal dependencies. Therefore they are not applied to static metabolomic spectra in a generic manner. In metabolomics, RNN architectures, such as long short-term memory (LSTM) and gated recurrent unit (GRU) networks, are implemented for time-resolved metabolomics, metabolic flux analysis, and dynamic perturbation experiments to capture delayed and non-linear metabolite responses across sampling time points. These models are particularly useful for predicting treatment response trajectories, circadian metabolic oscillations, and disease progression based on longitudinal metabolomic measurements. These algorithms learn and extract unique elements for pattern categorization and regression. These AI methods can also highlight candidate metabolites for drug targeting and evaluate compound effects in silico, supporting early-phase drug development. Reinforcement learning algorithms may guide the design of a metabolomics study. These algorithms learn from known data and issue recommendations on sample selection and desired measurements with an eye toward efficient resource utilization.

### 4.3. Limitations of Metabolomics Tools and Strategies for Data Integration

Although many robust data management tools and new applications for data utilization have been discussed in this review, many limitations of these metabolomics tools still need to be addressed [5,6]. First, the set of compounds which can be targeted is limited. Similarly, compounds that are not targeted will remain undetected. Increasingly complex diagnostic tools are in development that permit detection of all possible trackable components in the sample, a significant improvement from serial single-metabolite analysis. There is further complexity in sample processing when targeted compounds require purification for proper calibrated detection and compounds of interest must be preprogrammed for machine recognition. Other limitations include low throughput speeds. Having corroborative information accessible from alternate sources such as genomics or other ‘omics may help mediate these issues. Further limitations lie in streamlining real-time detection, which is exacerbated by the complexity of the multistep process [120].

While high-dimensional untargeted metabolomics combined with machine learning has become pervasive in discovery research, this “many metabolites × ML” paradigm carries inherent challenges that merit critical evaluation. Untargeted approaches generate very large feature spaces that often require complex statistical and computational methods to interpret. The identities of many detected signals remain unknown due to gaps in spectral libraries and reference standards, complicating downstream biological interpretation [121]. Machine learning models trained on such extensive datasets may tend to overfit when sample sizes are limited and can function as “black boxes.” This makes it difficult to extract actionable mechanistic insight or validate causal relationships. These are limitations that constrain clinical adoption [122]. In contrast, targeted metabolomics assays focus on a defined subset of known metabolites, enabling absolute quantification, improved reproducibility, and clearer biological interpretation, thereby facilitating clinical validation and regulatory acceptance [123]. Targeted methods are routinely used to validate candidate biomarkers identified in untargeted screens, monitor specific pathways, and quantify low-abundance metabolites with high precision [15]. Biologically informed feature selection and dimensionality-reduction strategies can reduce the complexity of ML models. These capabilities enhance interpretability by prioritizing metabolites with known relevance to physiological pathways. This strategy is increasingly discussed in the literature as providing more robust and clinically meaningful metabolomics analyses [124]. Together, these considerations emphasize that while high-dimensional ML is a powerful discovery tool, integrating it with targeted, biologically constrained, and platform-optimized approaches advances metabolomics toward reproducible, interpretable, and clinically actionable outcomes.

High-throughput analysis can be extremely helpful in the intraoperative setting, such as evaluating tissue margins, tissue composition and tissue pathology or in the critical care setting. It is important to balance the cost-effectiveness of various evaluation techniques such as ELISA and immunochromatography given systemic economic constraints.

Since metabolomics data can involve thousands of metabolites, the high dimensionality of data can make it difficult to achieve statistical power. In the preprocessing stage, there is further risk of instrument drift and sample processing variation. This can often be improved with data normalization and batch effect correction. Integrating this data with other genomic and proteomic markers and their associated variability and potential biases in measurement and processing can bring additional complexity. Statistical methods can also vary with attendant risks of limited knowledge of metabolite behavior and overfitting of data. Univariate, multivariate statistics and machine learning approaches may be used and cross-referenced with data from known metabolite databases to identify metabolites’ functional and regulatory roles. Decision tree and random forest models are commonly used for feature selection by ranking metabolites according to their predictive importance. In contrast, unsupervised clustering approaches (e.g., hierarchical clustering and k-means) are applied as a separate analytical step to group metabolites or samples based on similarity in their abundance profiles.

#### Platform-Specific Machine Learning Challenges in Metabolomics

While machine learning algorithms are widely applied in metabolomics, their performance is strongly influenced by platform-specific data characteristics [125]. In NMR-based metabolomics, relatively low analytical sensitivity and extensive spectral overlap limit the number of reliably detected metabolites. They can also introduce multicollinearity, which may reduce feature stability and compromise model interpretability. These limitations necessitate spectral deconvolution, region exclusion, and peak alignment strategies prior to model training [84]. In mass spectrometry-based metabolomics, additional challenges arise from adduct formation, in-source fragmentation, batch effects, and annotation uncertainty. Multiple ion species for a single metabolite inflate feature dimensionality and introduce redundancy, which can create bias in feature importance estimates in tree-based and regression-based models. Uncertainties in compound annotation propagate into training labels and can degrade supervised learning performance [84]. Missing values are prevalent in metabolomics due to detection limits and stochastic ion suppression. As a result, advanced preprocessing strategies, such as probabilistic quotient normalization, LOESS-based signal correction, k-nearest neighbor and random forest-based imputation along with batch effect correction are essential for preserving biological signal and ensuring reliable downstream machine learning performance. Inadequate normalization and imputation can substantially alter classification accuracy, biomarker ranking, and pathway enrichment outcomes, highlighting the critical importance of preprocessing pipelines in metabolomics-specific ML workflows [101].

### 4.4. The Future of Metabolomics Processing and Data Analysis

Future metabolomics tools will increasingly integrate AI to improve throughput, identify druggable pathways, and accelerate translational research. Open-source, robust, and user-friendly software platforms will make it easier to link metabolomic insights to therapeutic development, supporting precision medicine and drug discovery. By analyzing complex datasets, these tools can pinpoint key elements of cellular processing and track sequential metabolic byproducts, revealing patterns that suggest potential therapeutic targets. While many existing tools focus primarily on annotations, challenges remain in processing LC-MS/MS and multi-omics data efficiently. Web-based platforms, often with associated GitHub repositories, provide growing datasets that improve model accuracy over time. Programming languages such as R, Python, and Java are increasingly integrated, enabling diverse datasets to be combined and analyzed more effectively. As these tools become faster, more computationally efficient, and increasingly robust, researchers will be able to uncover meaningful metabolic patterns more reliably, directly translating software improvements into actionable insights for drug discovery and translational research.

### 4.5. Ethical and Privacy Considerations in Clinical AI

While this review identifies technical limitations such as high dimensionality and statistical power, the clinical implementation of AI-driven metabolomics also faces significant privacy and ethical challenges. As workflows move toward individualizing diagnosis and treatment, the datasets generated contain sensitive biological information. Ensuring data security is critical, particularly when integrating multi-omics data (genomics, proteomics, and lipidomics) that could potentially identify individual participants. Future research must prioritize the development of privacy-preserving AI architectures to ensure that the transition from ‘metabolomic insight’ to ‘translational therapeutic discovery’ remains compliant with ethical standards and regulatory frameworks.

### 4.6. Critical Evaluation of AI and Multi-Omics Integration

Although AI-enhanced workflows and multi-omics integration are frequently presented as key innovations in metabolomics, several challenges and trade-offs limit their mechanistic insight and scientific impact. Multi-omics datasets are heterogeneous and often incomplete, complicating integration and interpretation of results. Rigorous preprocessing and careful handling of missing data are important to avoid introducing bias or obscuring biological signals [126]. Untargeted metabolomics produces extensive raw data, including many unidentified features. There is limited coverage of comprehensive spectral libraries, and uncertain feature annotation weakens AI models’ ability to map computational patterns to actual biochemical mechanisms [127]. Machine learning models applied to high-dimensional metabolomics frequently face the curse of dimensionality, in which the number of variables exceeds the number of samples, increasing the risk of overfitting and reducing generalizability across cohorts [127]. Deep learning and other complex models may achieve high predictive performance but often lack transparency, making it difficult to interpret which metabolic features drive model outputs and how these relate to underlying biology. Similarly, multi-omics integration holds promise for linking metabolism with upstream regulatory layers, but differences in data scale, noise profiles, and statistical properties across omics types remain obstacles to reliable integration and mechanistic inference [128].

## 5. Conclusions

The integration of AI-driven automation into metabolomic workflows represents a transformative shift in our ability to interpret the complex biochemical interactions of living organisms. By synchronizing diverse software toolkits, from preprocessing to post-analysis, researchers can create a more comprehensive “workflow engine” that transforms high-dimensional data into actionable biological insights. This approach moves beyond simple metabolite identification to a dynamic understanding of how interventions alter systemic health and disease states.

Ultimately, the synergy between AI and multi-omics strategies is the key to bridging the current gap between basic metabolomic research and translational therapeutic discovery. By addressing the challenges of high-throughput data analysis and providing a scalable framework for biomarker discovery, these computational approaches provide the foundation for the next generation of precision medicine and individualized clinical interventions.

## Figures and Tables

**Table 11:** Machine Learning and Deep Learning Algorithms in Metabolomics Data Analysis.

Algorithm	Strengths	Weaknesses	Applications in Metabolomics	Metabolomics Tools
Random Forest	High accuracy and robustness to overfitting	Slow training and prediction times	Identifying biomarkers in disease diagnosis	MetaboAnalyst, KPIC2, W4M
Support Vector Machine	Good at handling high-dimensional data	Sensitive to outliers	Classifying samples based on metabolic profiles	MetaboAnalyst, apLCMS
Neural Network	High degree of accuracy, able to manage large datasets	Requires large data amounts for training	Identifying metabolic pathways and prediction of metabolic fluxes	CRANK-MS, MetaboLabPy
Deep Belief Network	Good at feature extraction and unsupervised learning	Requires large data amounts for training	Identifying unknown metabolic compounds and clustering of samples based on metabolic profiles	Custom Python/R pipelines
Convolutional Neural Network	Good at image recognition and handling high-dimensional data	Requires large data amounts for training	Identifying metabolic images and prediction of metabolic fluxes	DeepMet, SIRIUS (for CSI:FingerID)
k-Nearest Neighbors	Simple and easy to implement	Sensitive to the choice of k and the presence of outliers	Classifying samples based on metabolic profiles	MetaboAnalyst, MZmine
Principal Component Analysis (PCA)	Good at dimensionality reduction and visualization of data	Can lose important information during dimensionality reduction	Identifying metabolic pathways and clustering of samples based on metabolic profiles	MetaboAnalyst, XCMS, GNPS
Linear Discriminant Analysis (LDA)	Good at feature extraction and classification	Assumes that the data maps to a Gaussian distribution	Identifying biomarkers in disease diagnosis and classification of samples based on metabolic profiles	MetaboAnalyst (as PLS-DA)
Artificial Neural Network (ANN)	High degree of accuracy, able to manage large datasets	Requires large data amounts for training and can be sensitive to overfitting	Identifying metabolic pathways and prediction of metabolic fluxes	CRANK-MS, MetaboAnalyst (via R)
Recurrent Neural Network (RNN)	Good at handling sequential data	Requires large data amounts for training and can be sensitive to overfitting	Identifying metabolic pathways and prediction of metabolic fluxes in time-series data	Custom time-series scripts

## Data Availability

The original contributions presented in this study are included in the article. Further inquiries can be directed to the corresponding author(s). Availability of Data and Materials (all web accessed on 15 September 2023): QC Tools (Table 2): https://github.com/bihealth/metaquac https://github.com/NBDZ/dbnorm https://cran.r-project.org/src/contrib/Archive/MetaClean/ https://github.com/bihealth/ Annotated Tools (Table 3): https://messar.biodatamining.be/ https://smart.ucsd.edu/classic https://academic.oup.com/bioinformatics/article/36/12/3913/5809525 https://bioinfo.cristal.univ-lille.fr/nrpro/ https://www.metabolomicsworkbench.org/data/analyze.php https://bio.informatik.uni-jena.de/software/canopus/ https://github.com/mohimanilab/molDiscovery https://github.com/agnesblch/MetIDfyR https://github.com/biocore/q2-qemistree https://ccms-ucsd.github.io/GNPSDocumentation/fbmn-iin/ https://github.com/pcastellanoescuder/FOBI_Visualization_Tool https://github.com/ccdmb/BioDendro https://github.com/ZhuMetLab/AllCCS http://allccs.zhulab.cn https://binner.med.umich.edu/ https://github.com/eMetaboHUB/MS-CleanR https://www.retip.app/ https://github.com/UofUMetabolomicsCore/QSRR_Automator/releases/tag/v1_exe https://github.com/skschum/MFAssignR https://github.com/HuanLab/McSearch https://redu.ucsd.edu/ https://masst.ucsd.edu/ https://npclassifier.ucsd.edu/ https://github.com/rickhelmus/patRoon http://lipidmaps.org/lipidlynxx/ Preprocessing Tools (Table 4) https://github.com/rendju/CROP https://github.com/ChiungTingWu/ncGTW https://github.com/griquelme/tidyms https://github.com/crmclean/Autotuner https://shiny.maths.usyd.edu.au/hRUV/ https://github.com/hasaniqbal777/MetumpX-bin Statistical and Visualization Tools (Table 5) https://github.com/amergin/epimetal http://epimetal.computationalmedicine.fi/ https://mpietzke.shinyapps.io/AutoPlotter/ https://github.com/cfbeuchel/Metabolite-Investigator https://viime.org/#/ https://bioconductor.org/packages/release/bioc/html/struct.html https://www.lipidr.org http://idrblab.cn/noreva/ https://doi.org/10.1371/journal.pone.0244013 https://github.com/fgcz/rawR https://github.com/Metaboverse https://github.com/optimusmoose/jsms https://github.com/emmag Platform Tools (Table 6) https://github.com/sipss/AlpsNMR https://github.com/BEKZODKHAKIMOV/SigMa_Ver1 https://github.com/stefhk3/nmrfilterprojects https://bitbucket.org/iAnalytica/mshub_process/src/master/ https://github.com/CCMS-UCSD/GNPS_Workflows/tree/master/mshub-gc/tools/mshub-gc/proc Database Tools (Table 7) https://coconut.naturalproducts.net http://metlin.scripps.edu/ https://github.com/cibionnmrlab/CSMDB-with-ConQuer-ABC https://curatr.mcf.embl.de/ Isotopic Tools (Table 8) https://github.com/jcapelladesto/isoSCAN https://md.tu-bs.de/ Lipidomics, MSI and Multomics tools (Table 9) https://github.com/dylanhross/lipydomics https://github.com/lifs-tools/lipidcreator https://github.com/LlucSF/Raman2imzML http://igc1.salk.edu:3838/summer/ https://bitbucket.org/salkigc/summer/src/master/

## Abbreviations

AI	Artificial Intelligence
MSCAT	Metabolomics Software Catalog
MS	Mass spectroscopy
NMR	Nuclear Magnetic Resonance
FTIR	Fourier-transform infrared
